# LDL-C plays a causal role on T2DM: a Mendelian randomization analysis

**DOI:** 10.18632/aging.102763

**Published:** 2020-02-10

**Authors:** Wenbin Pan, Weiju Sun, Shuo Yang, He Zhuang, Huijie Jiang, Hong Ju, Donghua Wang, Ying Han

**Affiliations:** 1 Department of Radiology, The Second Affiliated Hospital of Harbin Medical University, Harbin, China; 2 Cardiovascular Department, The First Affiliated Hospital of Harbin Medical University, Harbin, China; 3 College of Bioinformatics Science and Technology, Harbin Medical University, Harbin, China; 4 Department of Information Engineering, Heilongjiang Biological Science and Technology Career Academy, Harbin, China; 5 Department of General Surgery, Heilongjiang Province Land Reclamation Headquarters General Hospital, Harbin, China; 6 Cardiovascular Department, The Fourth Affiliated Hospital of Harbin Medical University, Harbin, China

**Keywords:** low-density lipoprotein cholesterol, type 2 diabetes mellitus, casual effect, Mendelian randomization, type 1 diabetes mellitus

## Abstract

Diabetic dyslipidemia is a common condition in patients with Type 2 diabetes mellitus (T2DM). However, with the increasing application of statins which mainly decrease low-density lipoprotein cholesterol (LDL-C) levels, clinical trials and meta-analysis showed a clearly increase of the incidence of new-onset DMs, partly due to genetic factors. To determine whether a causal relationship exists between LDL-C and T2DM, we conducted a two-sample Mendelian Randomization (MR) analysis using genetic variations as instrumental variables (IVs). Initially, 29 SNPs significantly related to LDL-C (P≤ 5.0×10^-8^) were selected as based on results from the study of Henry et al, which processed loci data influencing lipids identified by the Global Lipids Genetics Consortium (GLGC) from 188,577 individuals of European ancestry. While 6 SNPs related to T2DM (P value < 5×10^-2^) were deleted, with the remaining 23 SNPs without LD eventually being deemed as IVs. The combined effect of all these 23 SNPs on T2DM, as generated with use of the penalized robust inverse-variance weighted (IVW) method (Beta value 0.24, 95%CI 0.087~0.393, P-value=0.002) demonstrated that elevated LDL-C levels significantly increased the risk of T2DM. The relationship between LDL-C and Type 1 diabetes mellitus (T1DM) with this analysis producing negative pooled results (Beta value -0.202, 95%CI -2.888~2.484, P-value=0.883).

## INTRODUCTION

Type 2 diabetes mellitus (T2DM) has become a global epidemic and major public health concern of late. The worldwide prevalence in adults aged 20-79 years has risen to 8.8% in 2017 and is expected to increase to 9.9% by the year 2045, with the result being that 9.5 billion adults will have diabetes [1] (https://diabetesatlas.org/). Diabetes is a leading cause of cardiovascular disease and is associated with premature morbidity and mortality [2].

As one of the major conditions associated with DM, diabetic dyslipidemia, has been reported to be closely and causally related to the genesis and the progression of atherosclerosis [3–5]. Diabetic dyslipidemia is quite common among patients with T2DM, with a prevalence ranging from 75-82%, while only 10% of DM patients with type 1 diabetes tend to show dyslipidemia [6]. With regard to the CV risk associated with DM, an important factor involves controlling levels of the low-density lipoprotein cholesterol (LDL-C) below a certain value as recommended by current guidelines [7–10]. One approach to decrease LDL-C levels is with use of statins. However, results from clinical trials and meta-analysis have indicated a clear statins induced dose-dependent increase in the incidence of new-onset DMs, particularly in patients with abnormal carbohydrate homeostasis [11–17]. Moreover, it has been suggested that the risk of T2DM, as associated with statin therapy, can be attributed to a genetic predisposition for increased levels of LDL-C in patients with a lower incidence of T2DM [18–20]. Therefore, it is important to determine whether a causal relationship exists between LDL-C and T2DM.

**Figure 1:**
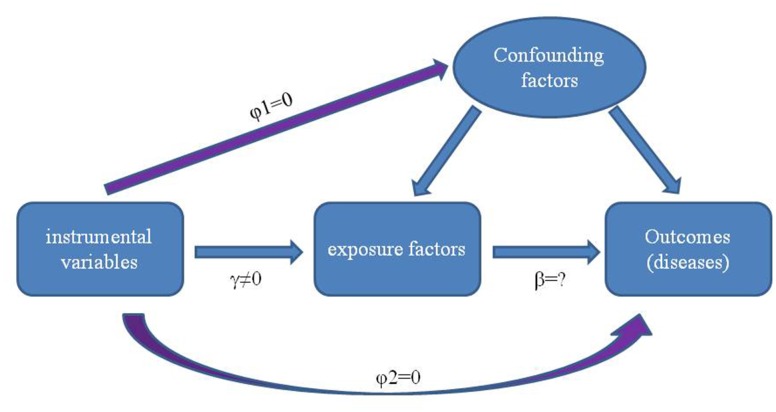
**Principles of using genetic variants as instrumental variable to estimate the causal influence of exposure factors on disease.** There is a strong correlation between genetic variation and exposure factors (γ≠0), and the genetic variation is independent of the confounding factors affecting the relationship between “exposure factors -outcomes” (φ1=0). Furthermore, genetic variation can only affect the outcomes through exposure factors but not other paths (φ2 = 0).

Use of randomized controlled trials to reveal causality can be problematic. In particular, these involve time-consuming procedures and are prone to confounding, reverse causality (i.e. disease processes affect exposure) and various other biases, which hinder, if not mislead, conclusions regarding associations between exposure factors and disease etiology [21, 22]. Therefore, incorporating the natural randomization inherent in the generation of genetic individuality, as can be accomplished with use of the Mendelian randomization (MR) method, provides a useful complement to traditional epidemiological studies [23, 24]. MR uses genetic variants as an instrumental variable (IV) to estimate and assess casual relationships between exposure of interest and outcomes [25–28] (Figure 1).

A major asset of the MR method is that genetic variation produces differences between individuals that influence health outcomes that are not affected by confounding or reverse causal bias which may distort the observations [24, 29, 30]. Moreover, the ability to combine large numbers of genetic variant data from genome-wide association studies (GWASs) with data from large numbers of disease outcome GWASs and various of databases enables MR to conduct comprehensive investigations as achieved with use of the two-sample MR analysis method [30–33].

Therefore, the goal of this report was to assess causal effects of LDL-C as related to the risk of T2DM using the two-sample MR approach. The causality between LDL-C and type 1 diabetes Mellitus (T1DM) was also investigated as a means to verify whether LDL-C demonstrates a specific causal relationship with T2DM or a more generalized relationship with all subtypes of diabetes.

## RESULTS

### IVs chosen for analysis

There were 29 SNPs that were significantly related to LDL-C (P≤ 5.0×10^-8^) as based on Henry et. al.’s study [35] in Set1, while 6 SNPs related to T2DM (P value < 5×10^-2^) were deleted and 23 SNPs were remained in Set2. As these 23 SNPs were assessed to be without LD, they were deemed as IVs in Set 3. Information on each of the 23 SNPs selected for analysis, in particular Beta coefficients of the SNP on the risk of LDL-C and T2DM and SEs, are listed in Table 1.

**Table 1:** Information on each of the 23 SNPs.

**SNP**	**phenotype_Beta**	**phenotype_SE**	**disease_SE**	**disease_Beta**
rs267733	0.0331	0.0053	0.019802627	-0.020408163
rs2710642	0.0239	0.0038	0.009950331	0.015306122
rs10490626	0.0508	0.0069	0.009950331	-0.030612245
rs2030746	0.0214	0.0038	0.009950331	0.015306122
rs1250229	0.0243	0.0042	0.009950331	0.015306122
rs7640978	0.0392	0.0069	0	-0.030612245
rs17404153	0.0336	0.0054	0	-0.020408163
rs4530754	0.0275	0.0036	0.019802627	0.015306122
rs4722551	0.0391	0.0049	0	0.025510204
rs10102164	0.0316	0.0045	0.029558802	0.015306122
rs4942486	0.0243	0.0037	0.009950331	-0.015306122
rs364585	0.0249	0.0038	0.009950331	0.015306122
rs2328223	0.0299	0.005	0.029558802	0.020408163
rs5763662	0.0767	0.0121	0.029558802	0.025510204
rs2479409	0.0642	0.0041	0.009950331	-0.015306122
rs1367117	0.1186	0.004	0.019802627	0.015306122
rs4299376	0.0812	0.0045	0.009950331	-0.015306122
rs3757354	0.0382	0.0044	0	-0.015306122
rs1800562	0.0615	0.008	0.019802627	-0.045918367
rs11220462	0.059	0.0059	0.019802627	0.015306122
rs8017377	0.0303	0.0038	0.019802627	0.020408163
rs7206971	0.0292	0.0055	0.009950331	0.015306122
rs6029526	0.0436	0.0052	0.019802627	0.015306122

SE, standard error.

### IVW results

**Figure 2:**
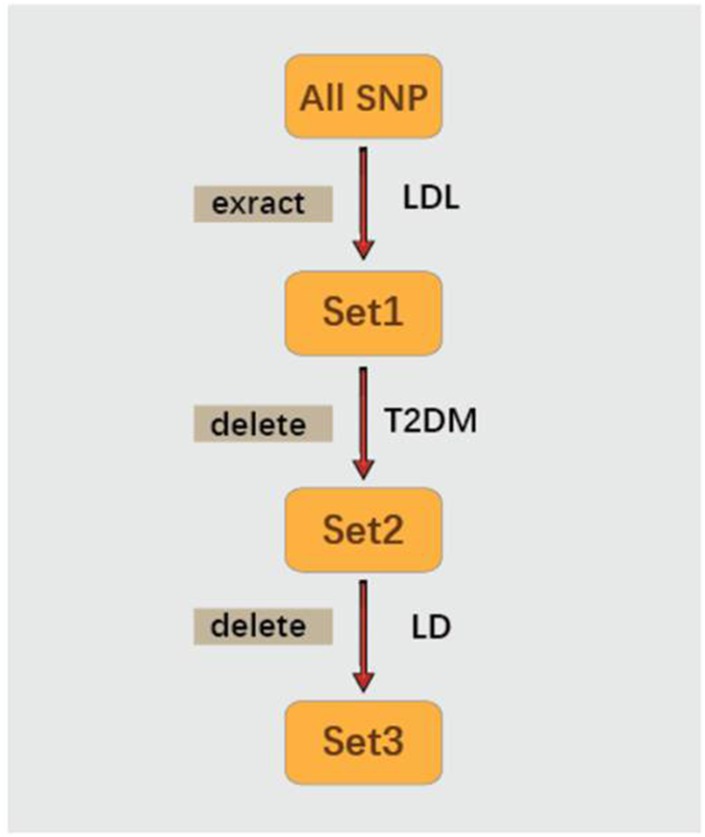
**Forest plot of the ORs and 95%CIs of the instrumental variables.**

Figure 2 contains the forest plot of estimates and 95% CIs representing the effect of each SNP of Set 3 on T2DM. The combined effect of all 23 SNPs on T2DM, as generated with the use of the IVW method, are shown in Table 2. The Beta value of elevated LDL-C associated with T2DM was 0.25 (95%CI 0.105~0.395, P-value=0.001). Similar Beta values were obtained using penalized IVW (Beta value 0.25; 95%CI 0.105~0.395; P-value=0.001), robust IVW (Beta value 0.24; 95%CI 0.087~0.393, P-value=0.002) and penalized robust IVW (Beta value 0.24, 95%CI 0.087~0.393, P-value=0.002) (Table 2). Taken together, these results demonstrate that elevated LDL-C levels significantly increased the risk of T2DM.

**Table 2:** The effect of LDL-C on T2DM estimated using IVW and MR-Egger methods.

**Method**	**Beta**	**Std error**	**95% CI**		**P-value**
IVW	0.250	0.074	0.105	0.395	0.001
Penalized IVW	0.250	0.074	0.105	0.395	0.001
Robust IVW	0.240	0.078	0.087	0.393	0.002
Penalized robust IVW	0.240	0.078	0.087	0.393	0.002
MR-Egger	0.062	0.146	-0.224	0.348	0.670
(intercept)	0.011	0.007	-0.003	0.025	0.135
Penalized MR-Egger	0.062	0.146	-0.224	0.348	0.670
(intercept)	0.011	0.007	-0.003	0.025	0.135
Robust MR-Egger	0.070	0.078	-0.082	0.222	0.367
(intercept)	0.010	0.006	-0.001	0.021	0.072
Penalized robust MR-Egger	0.070	0.078	-0.082	0.222	0.367
(intercept)	0.010	0.006	-0.001	0.021	0.072

CI, confidence intervals; IVW, inverse-variance weighted; LDL-C, low-density lipoprotein cholesterol;

OR, odds ratio; SE, standard error; T2DM, type 2 Diabetes Mellitus.

Table 3 shows the sensitivity analysis result of SNPs based on leave-one-out validation. The estimate have a great change after removing rs1367117 or rs11220462 but not after removing other SNPs. These results demonstrated that rs1367117 or rs11220462 drive the Penalized robust IVW estimate.

**Table 3:** The sensitivity analysis result of SNPs based on leave-one-out validation.

**SNP**	**Beta**	**SE**	**95%CI**		**P value**
rs267733	0.159	0.074	0.014	0.303	0.015
rs2710642	0.144	0.074	-0.001	0.289	0.032
rs10490626	0.153	0.074	0.008	0.298	0.023
rs2030746	0.144	0.074	0	0.289	0.032
rs1250229	0.144	0.074	-0.001	0.289	0.032
rs7640978	0.149	0.074	0.005	0.294	0.028
rs17404153	0.15	0.074	0.005	0.295	0.027
rs4530754	0.138	0.074	-0.008	0.283	0.035
rs4722551	0.15	0.074	0.005	0.295	0.027
rs10102164	0.129	0.074	-0.016	0.275	0.036
rs4942486	0.155	0.074	0.011	0.3	0.02
rs364585	0.144	0.074	-0.001	0.289	0.032
rs2328223	0.138	0.074	-0.007	0.283	0.032
rs5763662	0.136	0.075	-0.012	0.283	0.044
rs2479409	0.18	0.077	0.028	0.331	0.009
rs1367117	0.139	0.089	-0.036	0.314	0.084
rs4299376	0.196	0.08	0.04	0.352	0.005
rs3757354	0.153	0.075	0.007	0.299	0.026
rs1800562	0.152	0.074	0.008	0.297	0.023
rs11220462	0.131	0.077	-0.019	0.281	0.054
rs8017377	0.142	0.074	-0.003	0.287	0.033
rs7206971	0.144	0.074	-0.001	0.289	0.003
rs6029526	0.134	0.075	-0.013	0.281	0.044

CI, confidence intervals; SE, standard error.

Table 4 contains results from the combined effects of SNPs on T1DM. No statistically significant positive association was obtained between LDL-C and T1DM as revealed from the Beta (0.019, 95%CI -0.009~0.048, P-value=0.178), as well as from the robust IVW (Beta 0.014, 95%CI -0.003~0.031, P-value=0.099) and penalized robust IVW (Beta -0.202, 95%CI -2.888~2.484, P-value=0.883). Accordingly, LDL-C shows a specific relationship with T2DM, but not T1DM.

**Table 4:** The effect of LDL-C on T1DM estimated using IVW and MR-Egger methods.

**Method**	**Beta**	**Std error**	**95% CI**		**P-value**
IVW	0.019	0.014	-0.009	0.048	0.178
Penalized IVW	0.036	0.004	0.028	0.044	0.000
Robust IVW	0.014	0.009	-0.003	0.031	0.099
Penalized robust IVW	-0.202	1.370	-2.888	2.484	0.883
MR-Egger	0.014	0.021	-0.028	0.056	0.511
(intercept)	0.011	0.031	-0.050	0.073	0.716
Penalized MR-Egger	-0.006	0.048	-0.100	0.088	0.898
(intercept)	0.039	0.045	-0.049	0.127	0.383
Robust MR-Egger	0.011	0.011	-0.010	0.031	0.319
(intercept)	0.009	0.027	-0.044	0.062	0.729
Penalized robust MR-Egger	0.038	0.003	0.033	0.043	0.000
(intercept)	-0.091	0.009	-0.108	-0.074	0.000

CI, confidence intervals; IVW, inverse-variance weighted; LDL-C, low-density lipoprotein cholesterol;

OR, odds ratio; SE, standard error; T1DM, type 1 Diabetes Mellitus.

The pleiotropic effects of these SNPs (IVs) were further estimated with use of robust and Penalized robust MR-Egger analyses. Results from these analyses revealed a Beta value of 0.062 (95%CI: -0.224~0.348, P=0.135) with an intercept of 0.011 (95% CI -0.003 to 0.025, P = 0.135). These findings indicate that a potential horizontal pleiotropic effect could bias our estimates (Table 2).

### DISCUSSION

In this report, we utilized a two-sample MR approach to expose potential causal effects of LDL-C on the risk of T2DM. The 29 SNPs showing a significant correlation with LDL-C were extracted from results of the study by Henry et al [34] who reported causal effects between LDL-C and colorectal cancer. However, 6 SNPs related to T2DM were deleted. Data on the associations between SNPs and T2DM were sourced from diabetes genetics replication and meta-analysis (DIAGRAM) consortium. The remaining 23 SNPs without LD were then used as the IVs. Results from IVW and Penalized IVW methods, as well as from the robust IVW and Penalized robust IVW methods reveal that elevated LDL-C levels have a causal effect on the risk of T2DM.

Results from randomized controlled trials and meta-analysis had indicated that lipid lowering treatment may increase the risk of T2DM [12–18]. And, results from the longitudinal Framingham Heart Study, also suggested that low LDL-C levels were associated with T2DM. Accordingly, a notable relationship between LDL-C and T2DM was apparent, with lipid-lowering treatment being associated with increased risks of T2DM [35]. However, the overall average results from 10-year follow-up data (N = 1,819) in non-diabetic first-degree relatives of consecutive patients with T2DM 30-70 years old, not treated with lipid-lowering drugs, revealed that a higher LDL-C level was significantly associated with a higher risk of T2DM, independent of age, gender, fasting plasma glucose, waist circumference or blood pressure [36]. Therefore, based upon these findings, the role of LDL-C as a causal risk factor for T2DM remained uncertain.

Here, we utilize MR, a technique which can provide robust and reliable evidence, as a means for assessing the relationship between LDL-C and T2DM. A number of factors contribute to the strength of our findings. First, all of the data on SNPs as associated with LDL-C and SNPs as associated with T2DM were obtained from large-scale GWASs. Previous studies employing MR used only a single genetic variant and the association between a specific risk factor and disease were limited to a single study population. Recently, increasing use of genome-wide association studies (GWAS) have resulted in a large amount of genotype-phenotype association data and genotype-disease association data. Such data allows for numerous genetic variants identified from many exposures to be used in MR analysis, which enables the ability to acquire comprehensive information regarding associations between exposure factors and disease etiology [30].

Second, all of the 23 SNPs significantly related to LDL-C (P value < 5×10-8), but not related to T2DM, were extracted as the IVs. Swerdlow et al. [18] assessed associations between SNPs (rs17238484 and rs12916) of 3-hydroxy-3-methylglutaryl-coenzyme A reductase (HMGCR) and the prevalence and incidence of T2DM by meta-analysis in 223,463 individuals from 43 genetic studies. Their results indicated that the rs17238484-G allele seemed to be associated with a higher risk of type 2 diabetes (OR per allele 1.02, 95% CI 1.00-1.05) and the rs12916-T allele association was consistent (1.06, 1.03-1.09). Similar results were reported by Ference et al. [37]. They compared the effects of lower LDL-C cholesterol levels that were mediated by variants in proprotein convertase subtilisin-kexin type 9 (PCSK9), HMGCR, or both on the risk of cardiovascular events and risk of diabetes in 112,772 participants from 14 studies with use of the MR method. Variants in these two genes were associated with very similar effects on the risk of diabetes: OR for each 10 mg per deciliter decrease in LDL-C cholesterol was 1.11 (95% CI, 1.04 to 1.19) for PCSK9 and 1.13 (95% CI, 1.06 to 1.20) for HMGCR. Similar results were observed with regard to the risk for cardiovascular events. However, these studies only focused on genetic variants in HMGCR and PCSK9, which are the intended drug targets, rather than all SNPs related to LDL-C. In this way, they failed to fully explain the relationship between LDL-C and T2DM as was accomplished in our current study.

Finally, in this report we utilized the most recent methodological developments of MR, including Penalized IVW, Robust IVW and Penalized robust IVW methods, as sensitivity analyses to provide additional means for investigating any pleiotropic effects of the genetic variants. To serve as a valid instrument, genetic variants must satisfy the assumptions of a strong correlation with exposure but an absence of pleiotropic effects with the outcomes. However, due to the complexities of biological effects, pleiotropic effects of variants are often unavoidable. To avert such an eventuality it is necessary to evaluate the sensitivity of the results with use of sensitivity analysis for the hypothesis that the evidence is insufficient or even contrary to the situation. Therefore, we utilized robust IVW and Penalized robust IVW methods for the inference of consistent and robust casual estimations. Robust regression in an inverse-variance weighted method and a simple median of the causal estimates from the individual variants, have considerably improved Type 1 error rates compared with conventional methods in a wide variety of scenarios when up to 30% of the genetic variants are invalid instruments [38].

The advantage of MR analysis is that confounding factors should not be considered when using genetic variation as IVs, because genetic variation is free and not affected by confounding factors. In contrast, confounding factors can seriously affect the results of observational studies. Therefore, most of these observational studies should be adjusted for potential confounding factors. Another possible explanation for the differences in results generated in our current MR study from those of previous clinical trials and meta-analysis may be due to the relative short-term duration of the trials as opposed to lifetime exposures to natural genetic variation, and potentially undefined “off-target effects” of medical treatments on carbohydrate homeostasis [39]. In fact, in response to hypolipidemic drug-cholesteryl ester transfer protein (CETP) inhibitors, which do not detrimentally affect carbohydrate homeostasis, a lower incidence of new-onset DM was reported [40].

Here, we get the inconsistent results using the MR-Egger method. Different methods have their own advantages and disadvantages in terms of consistency of causal effect estimation and effectiveness. Thus, the causal relationship need to be verified using randomized controlled trial (RCT). The results of the MR-Egger method indicate that the selected SNPs may affect the outcome through other pathways, as conclusions from a single method may be somewhat one-sided. This possibility can only be verified with more reliable information, as there are no other statistical methods currently available which can explain whether the selected SNPs have pleiotropic effects. By comparing other disease phenotypes such as insulin resistance [41] and obesity [42], it does not appear that the selected SNPs affected T2DM through other pathways. Unfortunately, we cannot assess all potential associated pathways, therefore conclusions based on the known information have certain limitations. With future developments of new methods and the increased availability of information, it will be possible to verified whether our selected SNPs have pleiotropic effects.

Results from our two-sample MR approach for the association of LDL-C and T1DM were negative, which indicates that a causality between LDL-C and T1DM was not possible. Such findings are consistent with the phenomenon that patients with type 1 diabetes usually show no dyslipidemia [6].

There exist some limitations in our study. First, racial differences may contribute to inconsistencies in results. Notably, the data on SNPs as associated with LDL-C and T2DM were from European studies, which restricts definitive conclusions for other, non-European, populations. Second, it has been reported that some genetic variations such as those in HMGCR and PCSK9 are also related to changes in body weight and waist to hip ratios, which are known risk factors for new-onset DM [18, 43, 44]. Finally, as a result of unrecognized effects of genotypes on other risk factors, there exists the possibility of residual confounding.

In conclusion, with use of two-sample MR analysis, we established that elevated LDL-C levels were associated with an increased the risk of T2DM, but not T1DM. These findings provide strong evidence for the clinical application of lipid lowering drugs in patients with T2DM as a means to reduce their risk of cardiovascular disease.

## MATERIALS AND METHODS

### Data source

Single nucleotide polymorphisms (SNPs) deemed as IVs in the two-sample MR analysis must satisfy the following criteria for an IV: 1) must be related to the exposure of interest, 2) should be independent of known confounding factors and 3) are not directly related to outcomes due to known confounding factors (Figure 1).

SNPs associated with LDL-C were selected from Henry et al.’s study [34], where the potential causal relationship between lipid traits (total cholesterol, triglyceride, LDL-C and high-density lipoprotein) and risk of colorectal cancer were reported. The relationship between genetic risk scores for lipid traits and colorectal cancer risk was investigated using data from seven previously reported genome-wide association studies (GWAS) of colorectal cancer comprised of 9,254 colorectal cancer cases and 18,386 controls. However, previously identified SNPs were obtained from the Global Lipids Genetics Consortium (GLGC) [45]. This large-scale study examined loci influencing these lipids using genome-wide and custom genotyping arrays in 188,577 individuals of European ancestry, including 94,595 individuals from 23 studies genotyped with GWAS arrays and 93,982 individuals from 37 studies genotyped with the Metabochip array, and identified 157 loci associated with lipid levels at P < 5 × 10−8, including 62 new loci. In this study, we used: 1) SNPs associated at genome-wide significance (i.e. P≤ 5.0× 10^-8^), 2) excluded SNPs that were correlated (i.e. Pairwise r^2^ value ≥ 0.01), as extracted in the Henry et al.’s study. Data of SNPs-disease including SNPs-T2DM was sourced from diabetes genetics replication and meta-analysis (DIAGRAM) consortium [46]. This consortium is comprised of a group of investigators with shared interests in performing large-scale studies to characterize the genetic basis of T2DM as focused on samples from individuals of European descent. The initial use of DIAGRAM (DIAGRAM v1) enabled the combination of T2DM genome wide association (GWA) studies from the UK (WTCCC), DGI and FUSION groups [47–49]. An incremental meta-analysis (DIAGRAM v2 or DIAGRAM+) resulted in the addition of five other GWAS of European-descent samples (DGDG, KORA, Rotterdam, DeCODE and EUROSPAN for a total of 8,130 cases and 38,987 controls) together with extensive replication involving 20 other cohorts. In the recent meta-analysis (DIAGRAM v3), 12,171 cases and 56,862 controls were collected [50]. This data set was then used as the basis for the selection of SNPs for T2DM replication of the Metabochip custom array. Summary data from this analysis are available at http://www.diagram-consortium.org.

### SNPs selection

**Figure 3:**
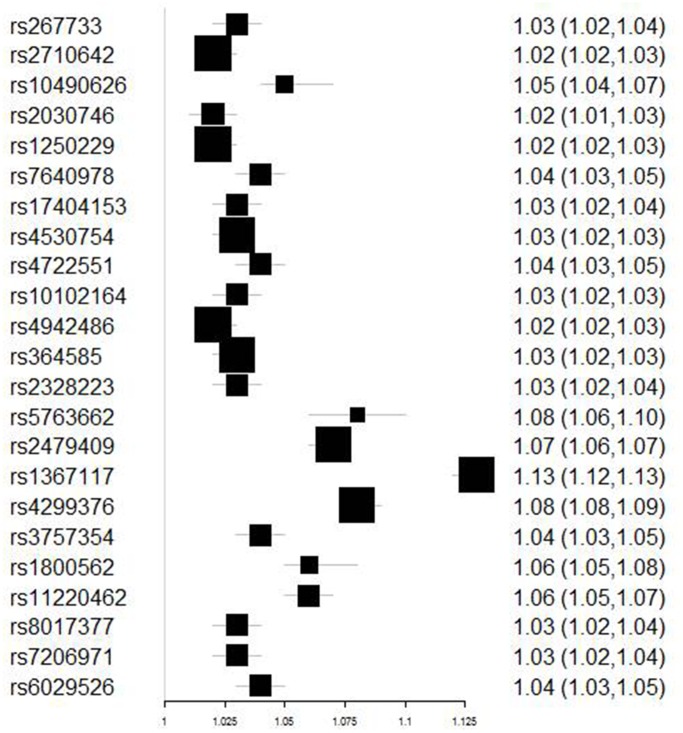
**The processes of SNPs selection.**

First of all, the SNPs which were significantly related to LDL-C (P≤ 5.0×10^-8^) as extracted from the Henry et al.’s study were categorized as Set1(Figure 3). Second, SNPs which were related to T2DM (P value < 5×10-2) were then deleted from Set 1. Remaining SNPs were categorized as Set2 (Figure 3). Finally, SNAP [51] (https://data.broadinstitute.org/mpg/snpsnap/) was used to eliminate linkage disequilibrium (LD). LD is a phenomenon in which two genes are transmitted simultaneously at different locations in a population significantly higher than that of the expected random frequency. The remaining SNPs without LD, categorized as Set 3, were then deemed as IVs for further MR analysis.

### Sensitivity analysis based on leave-one-out validation

The leave-one-out validation was performed to test the sensitivity of the selected SNPs (IVs). Each of SNP in IVs was removed from the IVs to carry out Pe nalized robust IVW estimate. And the fluctuation of the results before and after removing the SNP was observed as its sensitivity.

### Perform analysis

Then the two-sample MR procedure was performed, which is named inverse-variance weighted (IVW) method. The calculation of the odds ratio $\text{OR}=\frac{ad}{bc}$ (Table 5).Correspondingly, the standard error (SE) of OR is calculated to instruct the floating of OR, which is in $\text{SE}=\sqrt{\frac{1}{a}+\frac{1}{b}+\frac{1}{c}+\frac{1}{d}}$. All of OR satisfy the normal distribution, the suggestion of data, whose means equal 0 and the variance equal 1 approximately. Based on the above hypothesis, the 95% confidence interval (CI) can be calculated. The lower OR of CI is OR-1.96xSE, and the upper of it is shown by OR+1.96SE. The conversion of beta and OR appears by *β* = 1n *OR* to facilitate our research. The difference of Beta including two terms: Beta>0 shows that the effective allele is the risk gene, which suggest the result is the correct; while on the other hand, Beta<0 indicates the risk allele is non-effective gene, which instructs that the Beta must be its turn according the formula of OR. After we got the individual affect of each SNP (IV) on T2DM, further analysis including Penalized IVW, Robust IVW and Penalized robust IVW are also be used to reduce the sensitivity of methods to the influence of outlying variants and provide more robust estimates in large samples [38].

**Table 5:** Compositions for the calculation of the odds ratio.

	**effective alleles**	**Non-effective alleles**
cases	a	b
controls	c	d

All the statistical processes were performed through the R Package of meta-analysis (http://cran.r-project.org/web/packages/meta/index.html) and Mendelian Randomization (https://cran.r-project.org/web/packages/MendelianRandomization/) [52].
